# Comparison of Dental Caries Risk Assessment Using CaRisk- A Simple Mobile Based Application and WHO deft, DMFT Scores: A Cross Sectional Study

**DOI:** 10.30476/dentjods.2023.98075.2051

**Published:** 2024-06-01

**Authors:** Canty Sandra S, Aparna S, Parangimalai Diwakar Madan Kumar

**Affiliations:** 1 Dept. of Public Health Dentistry, Ragas Dental College and Hospital, Chennai, Tamil Nadu, India

**Keywords:** Caries Detector, Dental Caries, Early Diagnosis, Mobile Application, Risk Assessment

## Abstract

**Statement of the Problem:**

It is essential to address caries risk at an early stage for the prevention of dental caries. Mobile application CaRisk is designed in a particular way to self-assess the dental caries risk by the individual’s themselves.

**Purpose:**

The current study aimed to assess the dental caries risk among age groups 5-6 and 35-44 using self-assessment caries risk mobile application CaRisk and compare it with the deft and DMFT values.

**Materials and Method:**

This cross-sectional study was conducted in Chennai, India; to evaluate the risk of dental caries in children aged 5 to 6 and adults aged 35 to 44. The scores of the mobile application CaRisk and the decayed- extracted- filled teeth (deft)/ decayed-missing-filled-teeth (DMFT) caries risk assessment were evaluated. Descriptive statistics were performed. The risk category was determined by frequency. Chi-square analysis was done to determine whether the DMFT scores and the CaRisk mobile app were associated. The correlation was performed between the CaRisk mobile application and DMFT scores.

**Results:**

Association was found between the caries risk assessment score of the mobile application CaRisk and the DMFT and deft scores of the adults and children for both the age groups 5-6 and 35-44 years respectively and it indicates that it was found to be statistically significant. Pearson’s correlation was performed to assess the strength of association and R-values obtained for the age group 5-6 and 35-44 years respectively, which was statistically significant (0.892 and 0.840).

**Conclusion:**

This CaRisk mobile application scores correlate with the deft and DMFT scores and it is an effective self-diagnosis tool for assessing dental caries risk assessment. Further, it is suggested that the mobile application CaRisk should be tested among a huge population.

## Introduction

Dental caries also referred to as tooth decay, is one of the most prevalent diseases among children that can be prevented [ 1 ]. Dental caries is a significant public health issue [ 2 ]. In recent decades, dental caries had greatly decreased and it is still declining in populations, according to numerous reports from throughout the world [ 3 ]. The World Health Organization (WHO) estimates that dental caries affects 60–90% of schoolchildren worldwide, mostly in developing nations [ 4 ]. Globally, the prevalence of dental caries has declined, but the declines in high, middle, and low-income countries differ [ 5 ]. Over the past four decades, the prevalence and severity of dental caries in 5- and 12-year-old individuals have declined. The caries component is very high, with the lowest prevalence in 12-year-olds in high-income countries, which also had the lowest prevalence in 35- to 44-year-olds [ 5 ].In both permanent and deciduous teeth, the age-standardized prevalence of caries decreased by 3.6% and 3.0% respectively [ 6 ]. Africa had the lowest prevalence of early childhood caries worldwide, at 30%, followed by Europe, at 43%, America, 48%, Asia, 52%, and Oceania, 82% [ 7 ]. The global prevalence of dental caries throughout the world was found to be 45.7% in Africa, 54% in India, 21.3% in the Americas, 52% in China, 77% in Europe, and 65.24% in Middle Eastern countries [ 8 - 13 ]. 

Dental diseases are distinct in that they are incredibly common worldwide, do not get better if left untreated, and necessitate expensive, time-consuming professional treatment [ 2 ]. The dentistry profession has long taken satisfaction in initiatives that have decreased dental caries, such as the use of systemic and topical fluorides, toothpaste, sealants, dietary changes, oral health awareness campaigns, and dental care [ 3 ]. However, some people are still more likely to develop dental caries due to various risk factors [ 3 ]. Individual risk factors such as poor oral hygiene habits, a lack of knowledge and skills, and a poor diet have a significant impact on the development of caries [ 3 ]. These risk factors need to be thoroughly investigated, addressed, and modified to stop the development of dental caries [ 2 ]. Hence, caries risk assessment plays an important role in dental caries prevalence [ 2 ].

Caries risk assessment (CRA) is the clinical process of determining the probability that a certain patient will acquire dental caries in the future and is therefore a crucial factor in the decision-making process for effective dental caries prevention and management [ 14 ]. 

Cariogram, Previser, Caries Management by Risk Assessment (CAMBRA), American Dental Association (ADA), and American Academy of Paediatric Dentistry (AAPD) Caries-Risk Assessment Tool (CAT) are a few types of caries risk assessment used by dental professionals [ 15 ]. A recently simplified, chairside, non-invasive, four-point strategy called "Caries Risk Assessment for Treatment (CRAFT)" has been proposed for the management of caries based on risk assessment [ 16 ]. Many mobile-based applications, such as MI dentistry, Cariogram, and myRisk have been developed using current technologies to evaluate the caries risk [ 17 - 19 ]. However, the main drawback is that they require complex requirements related to plaque and saliva and it can only be evaluated by dental professionals [ 17 - 19 ]. There are many self-assessment and self-management applications for oral and breast cancer [ 20 - 21 ]. But there is no simple self-assessing application for dental caries, which becomes relevant in situations like the COVID-19 pandemic where dental care was predominantly based on an emergency basis [ 15 , 22 ]. The financial burden associated with treating dental caries has increased over the years. Especially people of lower socio-economic status and those living in rural areas would be unwilling to afford for these dental treatments. Therefore, early diagnosis of dental caries is essential [ 23 ]. This initiated us to develop the self-assessment caries risk mobile application CaRisk that was based on the ADA caries risk assessment form for 0-6 and more than 6 years old.

In a previous study conducted in our department, study participants lacked an understanding of terms such as fluoride exposure and dental home [ 24 ]. The use of the CaRisk mobile application in the current study was intended to address this gap by improving the study population's understanding by adding notes and pictures. 

To test the validity of this mobile application, content validation and face validation were performed. The results concluded that the caries risk assessment mobile application could provide an opportunity for self-assessment; it could be used to provide risk-based dental care [ 25 - 26 ]. Based on this, this study aimed to assess the dental caries risk using the self-assessment caries risk mobile application CaRisk and compare it with the decayed- extracted- filled teeth (deft) and decayed- missing- filled- teeth (DMFT) among the age group 5-6 and 35-44 years [ 27 - 28 ].

## Materials and Method

This cross-sectional study was designed to assess the dental caries risk among 5-6 years old children and 35-44 year old adults in Chennai city. The study was carried out from October - December 2022. A total of 200 people participated in this study from the church of Chennai city. A detailed study protocol explaining the objectives and methodology of the study was prepared and submitted to Institution Review Board, Ragas Dental College, and Hospital, Chennai – 600119, India. This study was initiated after obtaining ethical clearance. The participant's parents or legal guardians were asked for their informed consent after carefully reading the information sheet. They were also told that the information would be kept private and would only be utilized for research.

### Sample Size Calculation

Based on the results from a prior study conducted by Janakiram *et al*. [ 29 ] in 2018 the sample size was calculated using G*Power software after a primary validation investigation (version 3.0.10). The software was given the following inputs: The alpha error was set at 5% (0.05), with the study's power set at 80% (0.08) and the effect size at 0.4. In each group, the sample size was calculated as 100. The study had 200 participants who were chosen based on the convenience sampling method.

### Inclusion Criteria

The inclusion criteria were defined as (1) subjects owning mobile phones, (2) Participants of the age group 5-6 and 35-44 years, and (3)Participants and parents or caregivers who gave consent to participate in the study.

### Exclusion Criteria

The inclusion criteria were defined as (1) Participants with physical or cognitive limitations who were not able to self-evaluate i.e those people with disturbed state of mind and not willing to participate voluntarily in the study, and (2) participants who had any acute dental problems at the time of examination, which require immediate attention.

### Procedure

**Figure 1:**
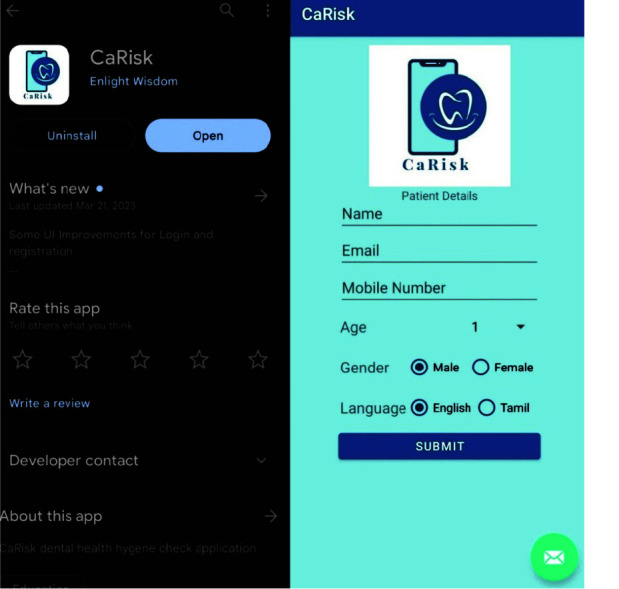
CaRisk mobile application in Google Playstore and its login interface

**Figure 2:**
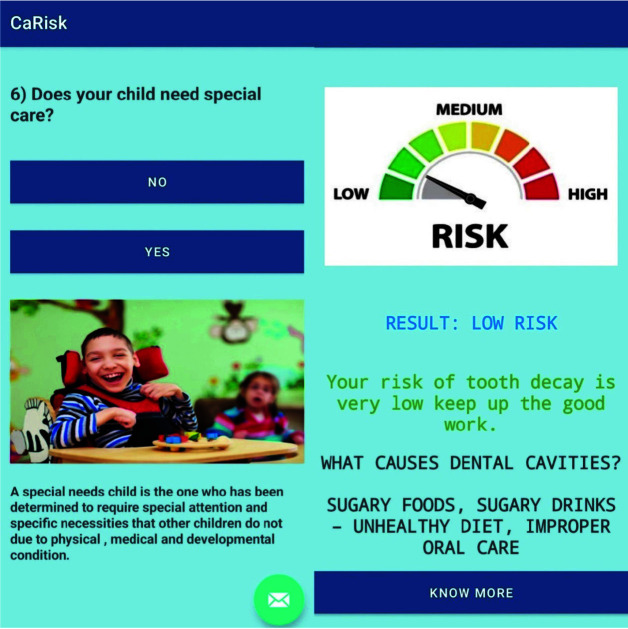
CaRisk question and out page

The CaRisk mobile application has a one-time password (OTP) login page followed by a layout page that displays the demographic details. The questions for the CaRisk mobile application were formulated based on the ADA caries risk assessment questionnaire (2009) for both age groups 0-6 and over 6 years in Tamil and English [ 25 - 26 ]. There were a total of 12 questions for the 0-6 year age group and 18 questions for more than 6 years age group. The questions were worded in such a way that non-medical professionals could understand them. Pictures and explanations were given for certain questions so that the questions could be better understood. Finally, the users will be displayed their caries risk. Lastly, users are shown their caries risk, with” A” representing low risk, “B” representing medium risk, and “C” representing high caries risk. In addition, oral hygiene instructions are displayed under the "Know More" section. The methodology of the mobile application is described in (Figures 1-2).

**Figure 3:**
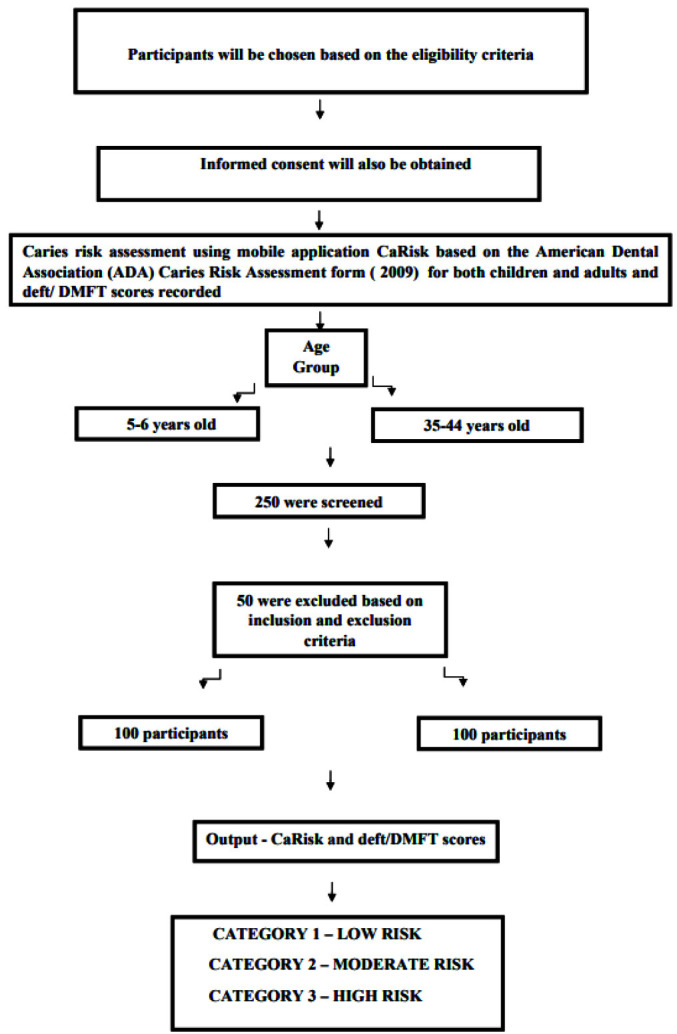
Flowchart illustrating the methodology of the study

A total of 200 individuals were assessed at the churches close to Selaiyur, Chennai, and 100 children and 100 adults who met the inclusion criteria were selected to participate in the study for the age ranges of 5 to 6 years and 35 to 44 years. Caries risk assessment was done using self-assessment caries risk mobile application CaRisk for 5-6 and 35-44 years. For the participants aged 5-6 years old, the consent was obtained from the parents or guardians and for the participants aged 35-44 years; the consent form was obtained directly. Only those subjects who gave consent to participate in the study were included in this study. All participants included in the study were sensitized regarding the CaRisk mobile application and the methodology of filling the same. For study subjects 5-6 years of age, the parents or guardians filled out the application based on the knowledge of their children and oral health status. Each participant approximately took 5 minutes to complete the application; they were able to assess whether their caries risk is low, medium, or high based on the input data. After completing the questions, the application showed whether they are at high, moderate, or low risk along with some instructions. After this, the principal investigator (Figure 3) recorded the DMFT and deft scores. DMFT and deft scores were calculated based on the WHO quantification [ 27 - 28 ]. For 5-6 year-old participants, the scores below 2.6 were classified as low risk, 2.7 -4.4 as moderate risk, and above 4.5 as high risk. For 35- 44 year-old participants, the scores below 9 were classified as low risk, 9-13.9 as moderate risk, and above 13.9 as high risk [ 27 - 28 ].

### Data Management

The data obtained will be entered into a Microsoft Excel Windows sheet and analyzed with IBM statistical pack age for social sciences (SPSS) software Version 26.

### Statistical analysis

Descriptive statistics were performed. The risk category was determined by frequency. Chi-square was performed to see the association between the CaRisk mobile application and DMFT scores. The correlation was performed between the CaRisk mobile application and DMFT scores.

## Results

**Figure 4:**
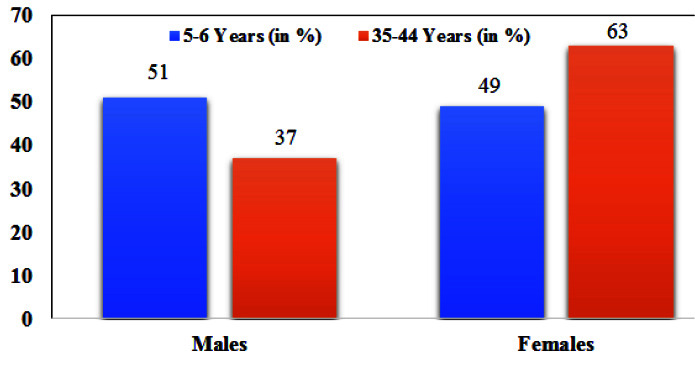
Percentage (%) of population based on gender

The mean age was 5.53±0.5 and 39.72±3.10 years. The percentage of males and females participated in the study was described in Figure 4.

**Figure 5:**
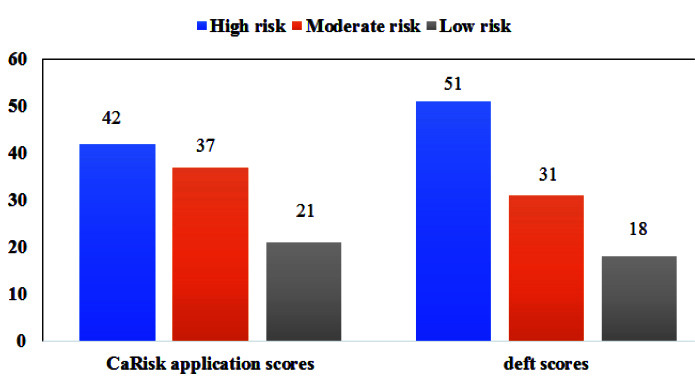
Risk assessment scores for 5-6 years age group

**Figure 6:**
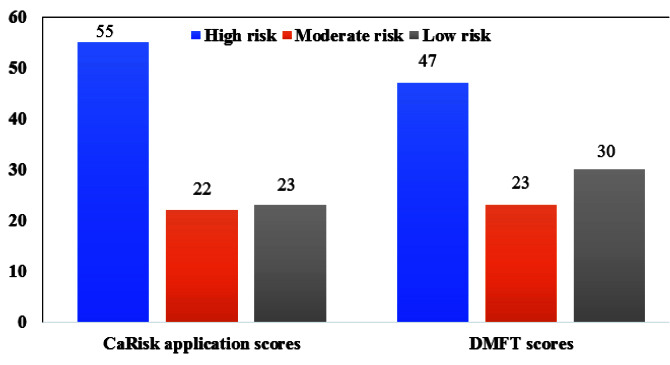
Risk assessment scores for 35-44 years age group

A trained professional to correlate the CaRisk application with the DMFT/deft scores assessed the outcome variables. The results of the risk assessment by mobile application CaRisk and DMFT/deft scores were described in Figure 5 and 6 [ 25 - 28 ].

The association was found between the caries risk assessment score of the mobile application CaRisk and the DMFT and deft scores of the adults and children for both the age groups 5-6 and 35-44 years respectively and it indicates that it was found to be statistically significant [ 25 - 28 ].

Pearson’s correlation was performed to assess the strength of association and R-values obtained were 0.892** and 0.840** for the age group 5-6 and 35-44 years, respectively, which was statistically significant.

## Discussion

In the present study, the CaRisk mobile application, which was based on the ADA risk assessment model, was used to determine caries risk in adults and children, and the risk assessment was compared with the DMFT caries risk assessment scores. In our study, our assessment was effective, especially in recognizing the high-risk patients in our group [ 25 - 28 ].

According to the results based on our mobile application CaRisk, over 42% of children and 55% of adults fit into the high-risk category, which, when compared to the DMFT and deft scores, revealed high risks of 51% for children and 47% for adults. The mean prevalence of dental caries in India for the age based on the WHO DMFT score for the age group 5 and 35-44 years were 49 % and 78% respectively [ 30 ]. The probable reasons for the high-risk category in our study of children might be the contributing factors such as the disregard for dental care, not brushing twice daily and family members who had dental decay in the previous six months. This study’s results almost match the deft scores in India [ 30 ].

Whereas for adults, consuming sugary content, having active dental carious lesions for more than 3 years, and not visiting the dentist regularly for dental care might be the factors for high caries risk. The CaRisk and DMFT scores were less when compared to the DMFT scores by WHO in India. The possible reason might be the study was conducted in the urban population and most of the populations were well educated.

Caries risk assessment is an important component of a dental examination. Numerous risk assessment models have been developed to identify individuals at risk for dental caries and highlight the key risk factors [ 31 - 32 ]. An ideal risk assessment model should have a high degree of accuracy in its prediction value, and be affordable, simple to use, and time-efficient [ 31 - 32 ]. Our dental caries risk assessment mobile application CaRisk satisfies all the above criteria.

In a pilot study by Anusha *et al*. [ 24 ] in India, caries risk was evaluated using the ADA caries risk assessment form for children between the ages of 6 and 10. In contrast to our results, the high risk of dental caries was around 57.2 %. This might be due to differences in the age group. The authors also reported low reliability (0.59). Lack of awareness among the survey participants about ideas like fluoride exposure and dental homes was one of the main reasons for this low rating [ 24 ]. The current study was based on this hypothesis and it made an effort to increase the study population's comprehension of these contributing factors by giving a thorough explanation in simple terminology and visualizing in the form of images using the mobile application CaRisk [ 24 ].

Giacaman AR *et al*. [ 33 ] in 2013 validated the findings of our investigation; they assessed the caries risk in adults and adolescents, recording 0.016% at low risk, 21.6% at intermediate risk, and 59.4% at high risk. The contributing factors were poor oral hygiene, dietary changes, a lack of dental awareness, and a general disregard for dental care. The results were consistent with the findings of our study [ 33 ].

 A systematic review conducted by Harris R *et al*. [ 34 ] in the year 2004, suggests that the frequency of consumption of sugar consumption was a risk factor for dental caries. In our study around 42 % and 55% from the age group 5-6 and 35-44 years of age selected the frequent between meal exposure option and hence; it is consistent with our study results. 

In a study conducted by Naik *et al*. [ 35 ] in 2018, regarding caries assessment using Cariogram, the average risk profile among government schoolchildren showed that the study participants had a 48% risk for caries development. The results were consistent with our study. Although the Cariogram model is truly comprehensive and shows how different caries-related factors affect a person's risk profile in relation to one another, the use of chair-side microbial tests, which are expensive and time-consuming, and salivary tests with microbiological cultivations, such as Mutans streptococci and lactobacilli enumeration, may delay the process from the perspective of the patient's motivation and may indicate its limited utility. The major disadvantage of using Cariogram is that they can be used only by professionals [ 35 ].

Arun *et al*. [ 36 ] in the year 2022, conducted the study to assess caries experience in the 3-to 60-year-old population of Rajasthan and to apply the newly derived mean scores for decayed, abraded, filled teeth and decayed, missing, filled teeth (deft/DMFT) in the Cariogram model to assess caries risk. Their cross-sectional study included 500 participants evenly divided into five groups (3-6, 7-12, 13-30, 31-44, and 45-60 years) [ 36 ]. Caries prevalence was highest (83%) in the 31-44 and 45-60 age groups and lowest (51%) in the 3-6 age groups. Caries experience scores increase with age, with the highest scores observed in the 31-44 and 45-60 age groups and the lowest in the 3-6 age group. The high caries risk category found for the age group 3- 6 years old was consistent with our study results [ 36 ].

In a study conducted by Palinee *et al*. [ 37 ] in 2022, developed a caries risk assessment application (myRisk app). Eighty-eight participants aged 12-29 years used the myRisk app [ 37 ]. According to myRisk, 7.9%, 71.6%, and 20.5% of participants were classified into the low-, moderate-, and high-risk groups, respectively. These results were in contrast with our study results. This might be due to the smaller sample size [ 37 ].

### Strength

The major strength of this study is that the results of this study correlate with the DMFT/deft scores indicating that it is a reliable tool for dental caries risk assessment. This mobile application CaRisk was developed for assessing dental caries risk by non-medical personnel or by individual patients themselves, in the absence of professional assistance or support. This self-assessing application can aid in the early diagnosis and prevention of dental caries in children so that more complex procedures like a root canal or dental implant therapy, can also be prevented. It displayed questions along with pictures and explanations, hence people were able to understand and comprehend in a better manner. This mobile application is also available in the Google Play Store.

### Limitation

The main limitation is that the research was done only to the people of the age group 5-6 years and 35-44 years. It is recommended to determine the sensitivity and specificity of the application on all age groups to ensure its usage on a larger scale. Another limitation of this study is that only those having smart mobile applications can use this mobile application and it is not on the iPhone Operating System (iOS) platform.

## Conclusion

It seems that CaRisk mobile application scores correlate with the deft and DMFT scores and it might be an effective self-diagnosis tool for assessing dental caries risk assessment. Only participants in the 5-6-year-old and 35-44-year-old age groups were included in the study. To ensure the application's widespread use, future studies must be conducted in large populations to assess caries risk in all age groups. 
